# Using System Dynamics to Understand Transnational Corporate Power in Diet-Related Non-communicable Disease Prevention Policy-Making: A Case Study of South Africa

**DOI:** 10.34172/ijhpm.2023.7641

**Published:** 2023-09-17

**Authors:** Penelope Milsom, Andrada Tomoaia-Cotisel, Richard Smith, Simon Moeketsi Modisenyane, Helen Walls

**Affiliations:** ^1^Department of Global Health and Development, Faculty of Public Health and Policy, London School of Hygiene and Tropical Medicine, London, UK; ^2^RAND Corporation, Santa Monica, CA, USA; ^3^College of Medicine and Health, University of Exeter, Exeter, UK

**Keywords:** Health Policy Process, Complex Systems, Corporate Power, Non-communicable Diseases, Commercial Determinants

## Abstract

**Background:** Complex interactions between political economy factors and corporate power are increasingly recognized to prevent transformative policy action on non-communicable disease (NCD) prevention. System science offers promising methods for analysing such causal complexity. This study uses qualitative system dynamics methods to map the political economy of diet-related NCD (DR-NCD) prevention policy-making aiming to better understand the policy inertia observed in this area globally.

**Methods:** We interviewed 25 key policy actors. We analysed the interviews using purposive text analysis (PTA). We developed individual then combined casual loop diagrams to generate a shared model representing the DR-NCD prevention policy-making system. Key variables/linkages identified from the literature were also included in the model. We validated the model in several steps including through stakeholder validation interviews.

**Results:** We identified several inter-linked feedback processes related to political economy factors that may entrench different forms of corporate power (instrumental, structural, and discursive) in DR-NCD prevention policy-making in South Africa over time. We also identified a number of feedback processes that have the potential to limit corporate power in this setting.

**Conclusion:** Using complex system methods can be useful for more deeply understanding DR-NCD policy inertia. It is also useful for identifying potential leverage points within the system which may shift the existing power dynamics to facilitate greater political commitment for healthy, equitable, and sustainable food system transformation.

## Background

**Key Messages**

**Implications for policy makers**

- Non-communicable disease (NCD) prevention policy-making involving stakeholders with competing interests and values is usefully understood as being embedded within a complex system of multiple inter-connected and interdependent elements.
- Within the diet-related NCD (DR-NCD) policy-making system, several inter-linked feedback processes involving different political economy factors and forms of power (instrumental, structural, and discursive) may be acting to further entrench corporate influence in policy-making over time, whilst other feedback processes may act to control it.
- Becoming ‘systems thinkers’ and ‘system doers’ can deepen health policy-maker understanding of the dynamics of DR-NCD (and other health) policy inertia and assist them in identifying strategic leverage points across the policy-making system for promoting more progressive policy change.

**Implications for the public**

Modern diets high in sugar, fat, salt and refined carbohydrates and containing a greater proportion of caloric sweeteners, vegetable oils, animal-sourced foods, and ultra-processed foods (UPFs)^1-3^ are considered key drivers of obesity and non-communicable diseases (NCDs) globally. So far most governments have failed to take policy action to adequately address the underlying drivers of unhealthy diets. This research suggests this inaction relates to various complex, inter-linked and interdependent feedback processes involving political and economic factors that may be further entrenching the power and influence of transnational food corporations over time. This research also identified other feedback processes that may act to control corporate power. Without adopting strategies to address corporate power in NCD prevention policy-making with significant system-level impact, it is likely that NCD prevalence will continue to rise globally contributing to reduced household incomes, restricted quality of life, premature death and deepening health and economic inequality.^4^

Globally there is well recognized transition towards diets that contain more sugar, fat, salt and refined carbohydrates and contain a greater proportion of caloric sweeteners, vegetable oils, animal-sourced foods, and ultra-processed foods (UPFs).^1-3,5^ This dietary trend is recognized as a key driver of obesity and diet-related non-communicable diseases (DR-NCDs) worldwide^1-3^ and is occurring at a faster rate in low-, and particularly middle-income countries than occurred in high-income countries due to rapid economic development, urbanization, and industrialization.^5,6^ UPFs, defined as ready-to-eat products, composed of substances derived from foods combined with cosmetic additives and derived from a series of industrial processes^7^ are particularly unhealthy. They include for example, most sugary drinks, confectionary, savoury snacks, baked goods, and sweet biscuits. UPFs represent an increasing proportion of people’s daily energy intake globally^3^ now contributing more than 50% of energy intake in high-income countries and up to 30% in middle-income countries where consumption is particularly on the rise.^8,9^

 These patterns of increased consumption have, in part, been driven by global market integration. This has been achieved through trade and investment liberalization (as well as technological developments) that has contributed to the production of larger volumes of UPFs with long shelf lives enhancing their tradability; promoted foreign direct investment by transnational corporations into food processing and retailing; and facilitated intensive global food marketing and advertising.^8,10-12^

 As populations increasingly undergo the nutrition transition, ensuring equitable access to healthy food and preventing DR-NCDs has been recognized as critical to achieving sustainable development.^13^ This is reflected in the Declaration of the United Nations (UN) Decade of Action on Nutrition 2016‐2025 and the 2018 UN Political Declaration on Prevention and Control of NCDs; and the inclusion of both nutrition and NCD targets within the Sustainable Development Goals. To achieve these targets, there have been repeated calls for government leadership and policy action that moves beyond abdicating responsibility for unhealthy eating to individuals and towards addressing the multiple food system drivers that create obesogenic food environments including in agriculture, trade, investment, public policy, and marketing.^12-16^

 Various frameworks and guidelines exist to inform such action.^15-17^ These include actions targeting the food supply (eg, removing sugar subsidies and implementing agricultural policies that incorporate health outcomes) and more directly the food environment (eg, taxes and import tariffs, healthier product reformulation, food standards in public institutions, banning unhealthy food marketing to children, targeted subsidies, and food labelling).^6,17^ However, the vast majority of governments have failed to translate these frameworks into policy action that adequately addresses the food system drivers of unhealthy diets, obesity and DR-NCDs.^6,18^ Further, minimal consideration has been given specifically to UPF in strategies aiming to reduce obesity or DR-NCDs.^3^

 As such, while a few countries have made progress on under-five obesity, the vast majority are off-track for meeting adult obesity and DR-NCD targets by 2025.^18,19^ Corporate influence, particularly from large transnational food corporations, and lack of political will have been identified as two key reasons for policy inaction.^13,18,20^ A small but growing body of public health policy literature seeking to explain these factors explores how political economy factors including political and economic actors, interests, institutions and ideas interact to limit political will and enhance corporate influence over DR-NCD prevention policy.^8,18,20-25^

 In line with a call for greater consideration of power in policy-making, more explicit analyses of how political economy factors shape power relations and inequities in DR-NCD policy-making are also emerging.^23,26,27^ For example, we recently explored how health harmful commodity corporations including tobacco, alcohol, and UPF corporations can exercise and benefit from different forms of power (instrumental, structural and discursive) via various mechanisms (eg, ideas, evidence, and institutions) to promote NCD policy inaction at the nexus of trade and health.^23^

 It has also only more recently been recognised that NCD prevention policy action/inaction occurs within a wider complex system of multiple inter-dependent political economy factors in feedback relationships.^18,23,27^ For example, one such feedback loop involves international trade and investment liberalization which has incentivized governments to promote and producers to deliver large volumes of commodities for export/use in global supply chains. The multinational agribusiness firms that have thrived under these conditions have, in turn, acted to maintain them, eg, by using the economic power gained for lobbying for trade policy that bring them financial benefit^28^ while entrenching limited nutrition policy space.

 Complex system problems involve multiple such feedback processes and, as such, are not caused by simple linear cause and effect relationships. Instead the behaviour of such ‘problem systems’ can vary over time (displaying dynamic complexity) making it difficult to predict the impact of interventions aimed at addressing the problem.^29^ Further, when dynamic complexity is not taken into account, proposed solutions to a complex system problem can be too limited in scope, low impact and generate unintended consequences over time.^30^

 Causal complexity in NCD prevention policy inaction is difficult to capture and analyze using traditional health policy process analysis methods.^31^ This has sparked an interest in applying systems thinking methods where policy action/inaction is understood as emerging from the dynamics of a wider political economy system.^13,27^ It involves mapping the interactions between multiple components within a complex system to understand how system behaviour changes over time.^32^ This may offer a promising tool for more reliably identifying impactful system-level solutions to NCD prevention policy inertia. However, with system methods only more recently being considered by public health policy process researchers, application of these methods in this research area remain limited. We identified just two studies that used system dynamics methods to explore the politics of food/nutrition policy-making. These were Baker and colleagues’ study exploring political commitment to ending malnutrition and how factors shaping nutrition actor network effectiveness can be strengthened^21^ and Clarke and colleagues’ use of systems thinking methods to explore the underlying dynamics of obesity prevention policy-making in Australia.^33^

 Further utilizing systems thinking approaches in this area, the aim of this work was to deepen understanding of the causal complexity of DR-NCD policy inaction due to multiple inter-dependent political economy mechanisms and different forms of transnational corporate power. Using South Africa as a case study, we apply system dynamics methods to develop several dynamic hypotheses to describe the problem of DR-NCD policy inaction. We then use these to identify potential leverage points in the system which may shift the existing power dynamics to facilitate greater political commitment for healthy, equitable and sustainable food system transformation.

## Methods

###  Systems Thinking and System Dynamics 

 A systems thinking approach facilitates the organization of complex information with a focus on the whole system.^34^ System dynamics, the systems thinking method used in this work, is based on a number of underlying characteristics of complex systems. These include that complex systems are made up of multiple interacting elements; these interactions drive system behaviour over time; relationships between elements are characterized by reinforcing and balancing loops; relationships between elements are also characterized by “stocks” and “flows” (eg, of resources, information or people); and cause and effect relationships change elements at different rates over time.^35,36^ The system dynamics process involves defining a problem preventing certain desired outcomes; qualitatively mapping the problem system structure; developing a dynamic numerical simulation model; testing different scenarios; and designing and comparing the effect of different policy options on key outcomes over time.^37^ This work undertakes the qualitative mapping step alone aiming to deepen understanding of the causal complexity of NCD prevention policy inaction.

###  Case Study Selection

 South Africa was selected as a case study for this work due to a combination of political, economic and health characteristics. Firstly, South Africa is a middle-income country that underwent a rapid period of trade and investment liberalization after Apartheid ended in 1994 and remains a relatively open economy to trade and investment. Secondly, South Africa’s geographic position and infrastructure makes it an attractive strategic hub from which UPF-producing corporations can develop new markets across Africa. This combined with South Africa’s recognition as a regional policy leader, may mean food corporations have particular interest in securing and maintaining a favourable regulatory environment in South Africa to prevent regional and continental policy transfer.

 At the same time, there has been significant growth in sales of UPFs and beverages in South Africa between 2006-2019.^8^ Along-side continuing high levels of underweight and nutritional deficiencies, the percentage of children and adults who are overweight or obese has significantly increased in South Africa in recent years, with a parallel increase in the per capita food supply of fat, protein and total calories.^38^ An estimated 68% and 31% of South African women and men respectively, are overweight or obese.^39^ Thirteen percent of children are overweight in South Africa,^39^ more than double the world average.^40^ In 2000, an estimated 36 504 deaths (7% of all deaths) in South Africa were attributed to excess body weight^41^ and overall, NCDs now account for 51% of all deaths annually.^42^

 However, while the South African government has adopted some internationally recommended policies to promote healthy eating, a number of DR-NCD prevention policies have yet to be adopted in the country and there remains significant incoherence between trade and investment policy and DR-nutrition objectives.^43^ This combination of factors allowed us to explore the dynamic complexity of how political economy mechanisms and corporate power may inhibit DR-NCD policy action over time.

 This study applied a participatory system dynamics modelling method using key stakeholder interviews to iteratively develop several initial causal loop diagrams (CLDs) hypothesizing how, over the past two decades of trade and investment liberalization, transnational corporate power may operate to weaken DR-NCD prevention policy norms in South Africa.

###  Data Collection 

 Semi-structured interviews with the offer of anonymity were selected as the method of data collection for this work (instead of group model building) for several reasons. These include the highly political nature of the topic area and highly unequal power relations between different policy actors which may limit frank discussion in a group model building setting.

 A stakeholder mapping exercise was initially undertaken to identify key policy actors with the assistance of a research collaborator within South Africa’s National Department of Health. Policy actors were selected purposively from the stakeholder mapping and then snow-ball sampling. Fifty key policy actors were invited for an interview from the following stakeholder groups: Department of Health (DH), Department of Trade and Industry (DTI), Department of Agriculture, Forestry and Fisheries (DAFF), National Treasury, intergovernmental organizations (IGOs), non-governmental organizations (NGOs) involved in supporting nutrition policy development, civil society groups (CSOs) involved in nutrition policy advocacy, academics with expertise in nutrition policy and/or the food system and food corporations. Twenty-nine policy actors agreed to take part in an interview, 13 did not respond and 10 declined the invitation (see Table 1). Four policy actors were however subsequently excluded since they did not provide in their interviews any explanatory data relevant for model building, resulting in 24 interviews with 25 participants ultimately being included. All government participants were Chief or Deputy Directors within their respective departments with one Deputy Director General. We attempted to conduct interviews with government stakeholders in both senior technical and more political roles (including Director Generals and Ministers), however it was extremely challenging to gain access to the latter group despite extensive attempts including via one of the investigators based within the DH. Industry representatives were governance and regulatory experts; and IGO, NGO, and CSO representatives had each been engaged in recent relevant nutrition policy processes in South Africa.

**Table 1:** Summary of Stakeholders Involved in Conceptual Model-Building

**Stakeholder Group**	**Key Stakeholders Invited to Participate**	**Key Stakeholders Interviewed**	**Stakeholders Included in Model Conceptualization**
DH	13	10	10
Health Attachés for South African Embassy in Geneva or Washington DC (current or past)	6	0	0
DTI	8	6	4
National Treasury	3	2	2
DAFF	3	2	2
NGOs/CSOs/IGOs	6	4	4
Academics	5	3	3
Industry	5	2	0
**Total**	**49**	**29**	**25**

Abbreviations: DH, Department of Health; DTI, Department of Trade and Industry; DAFF, Department of Agriculture, Forestry and Fisheries; IGOs, intergovernmental organizations; NGOs, non-governmental organizations; CSOs, civil society groups.

 Each policy actor participated in a semi-structured interview lasting on average between 45-75 minutes between May and September 2019. Interviews were conducted in-person in Cape Town or Pretoria or telephonically where in-person interviews were not possible. The interview guide was structured to elicit an in-depth understanding of key policy actors’ ideas, values, interests and positions in relation to nutrition and trade, investment and economic objectives; perceptions of the influences that trade and investment agreements and other trade and investment-related factors have on nutrition policy processes; and the strategic approaches adopted by stakeholders to achieve their desired nutrition or trade/economic objectives. Wherever possibly ‘why’ and ‘how’ questions were used during the interviews to get at the causality that participants perceived. All interviews were recorded and later transcribed in full and handwritten notes transferred into Microsoft Word documents.

###  Data Analysis

####  Individual Causal Loop Diagram Development 

 Data analysis was undertaken using purposive text analysis (PTA) to systematically identify causal statements from which linkages between system variables/elements could be identified to inform model conceptualization.^43,44^ In PTA coding is initially inductive, later also employing a deductive approach as a coding index develops during the text analysis process. Data interpretation and model conceptualization was also informed by a conceptual framework for analysing different forms (instrumental, structural, and discursive) and mechanisms of power in health policy-making that we previously developed, tested and refined in a related realist review.^23^ Details of the conceptual framework, including descriptions of the different forms and mechanisms of power, and how the framework was developed are included in Supplementary file 1.

 For each interview transcript all data segments describing a causal process were extracted and documented on a PTA coding chart. The cause variable, effect variable and the polarity of the relationship was then represented in a simple words and arrow diagram (See Supplementary file 2 for an example of a PTA coding chart).^43,44^ These were then merged into CLDs for each participant, representing each participant’s mental model defined as “a relatively enduring and accessible, but limited, internal conceptual representation of an external dynamic system.”^45^

 As PTA and CLD development progressed, standardised system variables were developed in an iterative process to include varied descriptions of the same causal phenomena by different participants in a single more generalized variable/relationship.^44^ Some causal relationships were also decomposed further by identifying implicit structures implied by the context (see Supplementary file 3 for examples of merging and generalizing variables and decomposing CLDs).^44^

####  Shared Causal Loop Diagram Development

 First, groups of two to four individual mental models (represented as CLDs) of participants with different perspectives on the same policy issue were composed. The individual CLDs in each group were then combined to generate seven shared CLDs based on different policy issues (eg, front of package food labelling, tax on sugar-sweetened beverages or marketing of breast milk substitutes). We then mildly ‘pruned’ the seven ‘policy issue’ CLDs – keeping delays and feedback structures but removing linear linkages.^43^ Next we combined the seven shared ‘policy issue’ mental models into final shared mental model (SMM) for all participants.^43^

 Details of the systematic approach to CLD combination can be found in Supplementary file 3 and in Milsom.^46^ While the majority of stakeholders (participants) provided additive rather than conflicting views, there were rare occasions where one or more stakeholders identified a relationship that another stakeholder expressly denied. In these instances, the relationship identified by the stakeholder with the closest experience of that part of the system was considered most accurate and was reflected within the final SMM.

 In a third step, the SMM was simplified and generalized for improved usability. This involved additional pruning^43^ of the SMMs to remove remaining linear linkages. Additionally, structures describing similar phenomena, but in more detail were aggregated into variables and relationships at a higher level of abstraction (Supplementary file 3 provides an example).^43,44^

 Now with a more detailed understanding of the interview data and broad sense of the overall system structure, it was possible to clarify certain parts of the model in an iterative process, moving from the two SMMs to the PTA coding charts and back. This included adding feedback processes that had not initially been obvious during the PTA and structures implied by the context but not explicitly mentioned in stakeholder interviews. For example, DH policy-makers did not specifically state that their increased focus on food/nutrition policies was due to increasing prevalence of DR-NCDs in South Africa, but this was considered a valid relationship to incorporate into the model structure (see sub-system I, DR-NCD prevalence **→** perceived salience of nutrition problem/impacts and solutions)given the government’s increased focus on food/nutrition policy following data illustrating a progressive increase in diet-related NCD prevalence.

 Finally, to further develop the SSM we drew on the findings of a related realist review,^23^ identifying two additional variables and nine linkages. These were initially included in the model to be reviewed for real-world relevance by stakeholders during model validation. At this stage due to the large number of variables and feedback loops, to improve usability of the SMM, it was divided into two distinct SMMs according to the forms of power described in the conceptual framework used in the analysis.^23^ The two SMMs were however highly inter-connected as indicated by a relatively high number of the same elements appearing in both sub-systems (eg, ‘strength of neoliberal beliefs, values and norms’ appears in both sub-systems).

###  Model Validation 

 We applied a series of validity tests to build confidence that the SMMs, as closely as possible, represented the aspects of the system that are relevant to the problem under study (the details of these can be found in Milsom^46^). The most important of these was to validate the resulting SMM via 90-minute structured dialogue sessions^47,48^ with eight key stakeholders previously interviewed (including representation from each of the stakeholder groups involved in model conceptualization, see Table 1). These stakeholders were local to South Africa and were known to have intimate knowledge of the system problem being examined. These sessions were conducted in November 2020. Each interview focused on presenting and discussing the model’s structure, behaviour and structure–behaviour connections.^48^ Participants were encouraged to question the real-world validity of the variables and feedback structures presented to them (particularly those added from the literature) and highlight flaws or missing structures.^48^ Detailed notes were taken by the interviewer (PM) during these sessions. Model structures not validated by stakeholders were removed and some parts that had not been fully understood from analysing the original interview data were clarified. Supplementary file 4 includes both prevalidated SMMs for comparison. Once the SMM was adequately revised to address the flaws and missing structures identified by stakeholders, it was considered to be the final conceptual model (a complex system model divided into two sub-systems of DR-NCD policy-making).^43^

## Results

 The conceptual model represents interactions between elements (eg, actions, conditions, and resources) that may explain observed limitations on DR-NCD policy progress over time despite increasing obesity and NCD prevalence in South Africa. The variables are connected via arrows representing causal links and form feedback loops – cycles of cause and effect that determine how the system’s behaviour changes over time.^36^ Reinforcing loops reinforce system behaviour over time and balancing loops regulate the effects of changes imposed on the system.^30^

###  Instrumental Power

**Figure 1:**
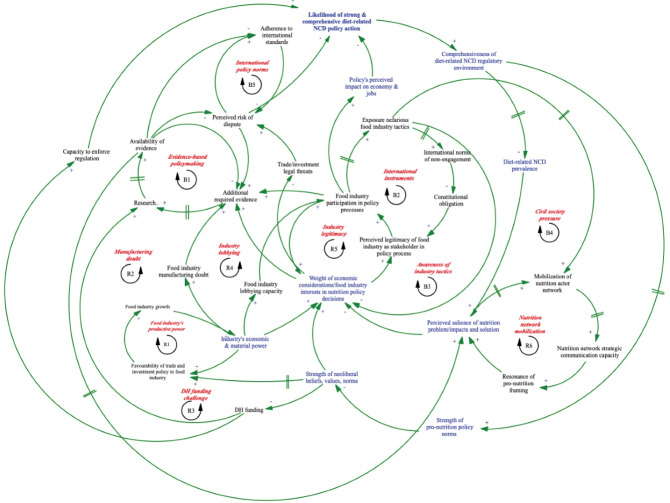
Sub-system I: Instrumental Power.

 Sub-system I (Figure 1) illustrates stakeholders’ understanding of the dynamic relationships between different mechanisms of industry’s intrumental power (see Supplementary file 1) over formal political decisions relating to DR-NCD policy. These mechanisms include relationships; knowledge and evidence; and rules.^23^ In this sub-system we identified six reinforcing loops and five balancing loops. Table 2 provides a key for both sub-systems.

**Table 2:** Key for Sub-systems I and II

**Figure:** 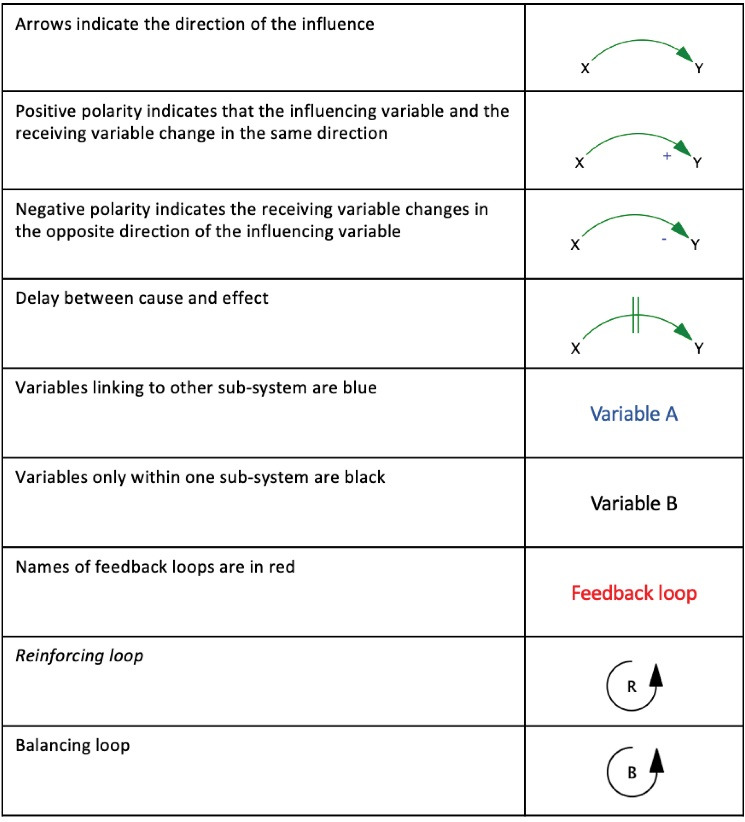

*R1 Food industry’s productive power:* Illustrates how as the food industry’s economic and material power increases, so too does the tendency for the government to adopt trade and investment policy favourable to them which, in turn, facilitates industry growth, further increasing their economic and material power.

*R2 Manufacturing doubt:* Economic and material power of industry increases the food industry’s ability to ‘manufacture doubt’ (for example, stakeholders reported infant formula companies fund biased child nutrition research and education) which increases the additional evidence required to support policy adoption. This often leads to significant delays in the policy process while the required research is gathered or conducted. When it is not possible to undertake research due to lack of resources or methodological challenges, the likelihood of policy adoption is very low with policy-makers frequently citing lack of evidence as the reason for policy inaction. Policy inaction and a weak regulatory environment then tends to perpetuate weak pro-nutrition policy norms, providing no counter force to policy norms which further expands industry economic power.

*R3 DH Funding challenge:* With increasing dominance of neoliberal beliefs, values and norms greater importance is placed on economy-focused departments (eg, the DTI) than health and departmental budgets reflect this. With relatively less funding, the DH’s capacity to conduct/commission DR-NCD policy research and to enforce regulations which both decrease the likelihood of DR-NCD policy action, again maintaining weak pro-nutrition policy norms which cannot challenge the predominant neoliberal ideology resulting in persistently low funding allocations to the DH.

*R4 Industry lobbying*: The food industry’s economic and material power increases their lobbying capacity, which expands their participation in nutrition policy processes, and potentially also the weight of industry interests in policy decisions. This in turn increases the level of evidence and advocacy effort required by the DH to advance a proposed regulation reducing the likelihood of DR-NCD policy action which, yet again, perpetuates weak pro-nutrition policy norms and the predominant neoliberal ideology. This drives a regulatory environment that is increasingly favorable to the food industry, supporting industry growth and in turn expanding their economic and material power and lobbying capacity.

*R5 Industry legitimacy*: Increasing dominance of the neoliberal ideology also drives policy-makers to weigh economic considerations more heavily in nutrition policy decisions leading to a heightened perception of food industry legitimacy as a stakeholder in the policy process which increases industry’s participation in the policy process (in turn further emphasizing the weight of economic considerations, and specifically industry interests, in policy decisions). Through their participation, industry is able to deepen the perceived economic impact of a given policy, generating greater resistance to policy adoption, yet again maintaining weak pro-nutrition policy norms which fail to counter the predominant neoliberal ideology.

*B1 Evidence-based policy-making:* The level of evidence required to support a proposed policy increases with increased food industry participation in the policy process, their ability to ‘manufacture doubt’ about a policy’s effectiveness, the weight of economic considerations and industry interests in nutrition policy-making and perceived risk of a trade/investment dispute. These factors drive evidence-based policy-making which, over time, drives research, increasing the availability of evidence (and likelihood of policy adoption) and in turn ultimately reduces the level of additional evidence required. Lack of local funding can significantly slow this process and delay policy adoption.

*B2 International instruments*: Industry participation in policy processes can also prompt public health advocates to expose the nefarious tactics used by industry to prevent policy adoption/promote their products, as for example occurred in the case of tobacco corporations. Over time, this kind of exposure reduces public and therefore political acceptability of industry and can lead to the development of international rules and norms institutionalized in legally binding international treaties, over-riding any domestic institutional obligation, and committing governments to restrict industry participation in policy processes. B2 can then provide a counter force to R5 and reduce industry participation and influence in policy processes.

*B3 Awareness of industry tactics:* When the food industry’s nefarious tactics are exposed (eg, non-adherence to pledges of self-regulation), nutrition policy-makers report lowering their consideration of industry interests during subsequent related policy-making.

*B4 Civil society pressure:*The exposure of nefarious industry tactics can also drive the mobilization of a nutrition actor network (external to government), ultimately increasing the perceived salience of a DR-NCD policy problem which can, once a certain threshold is reached, reduce the influence of economic and industry concerns in nutrition policy decisions. Both balancing loops B3 and B4 may provide important counter forces to R5’s ‘vicious’ cycle of increasing industry influence in policy processes.

*R6 Nutrition network mobilization:* Nutrition networks (including NGOs, academia, communication and advocacy experts, grassroots groups, and other governments and spanning from the local to the international) can lead to expansion of the network’s strategic communication capacity (eg, public education, targeted lobbying strategies for different policy actors and use of various advocacy/communication tools) which may increase the resonance of pro-nutrition issue framing and in turn increase the perceived salience of the issue and proposed solution among stakeholders, ultimately reducing the weight of economic considerations during decision-making.

 However, when their participation in policy processes is limited by such processes as described above, multinational corporations are more likely to use or convince other governments to use legal threats, including threats of international trade and/or investment disputes. Such threats increase perceived risk of a trade dispute.

*B5 International policy norms:* Health policy-makers ensure adherence to international standards to lower the perceived risk of a trade/investment dispute. This can become problematic if international standards limit the comprehensiveness of a proposed policy since they also have been influenced by industry interests. Perceived risk of a trade/investment dispute also drives *B1 evidence-based policy-making,* further sustaining the evidence-based policy-making approach.

###  Sub-system II: Structural and Discursive Power

**Figure 2:**
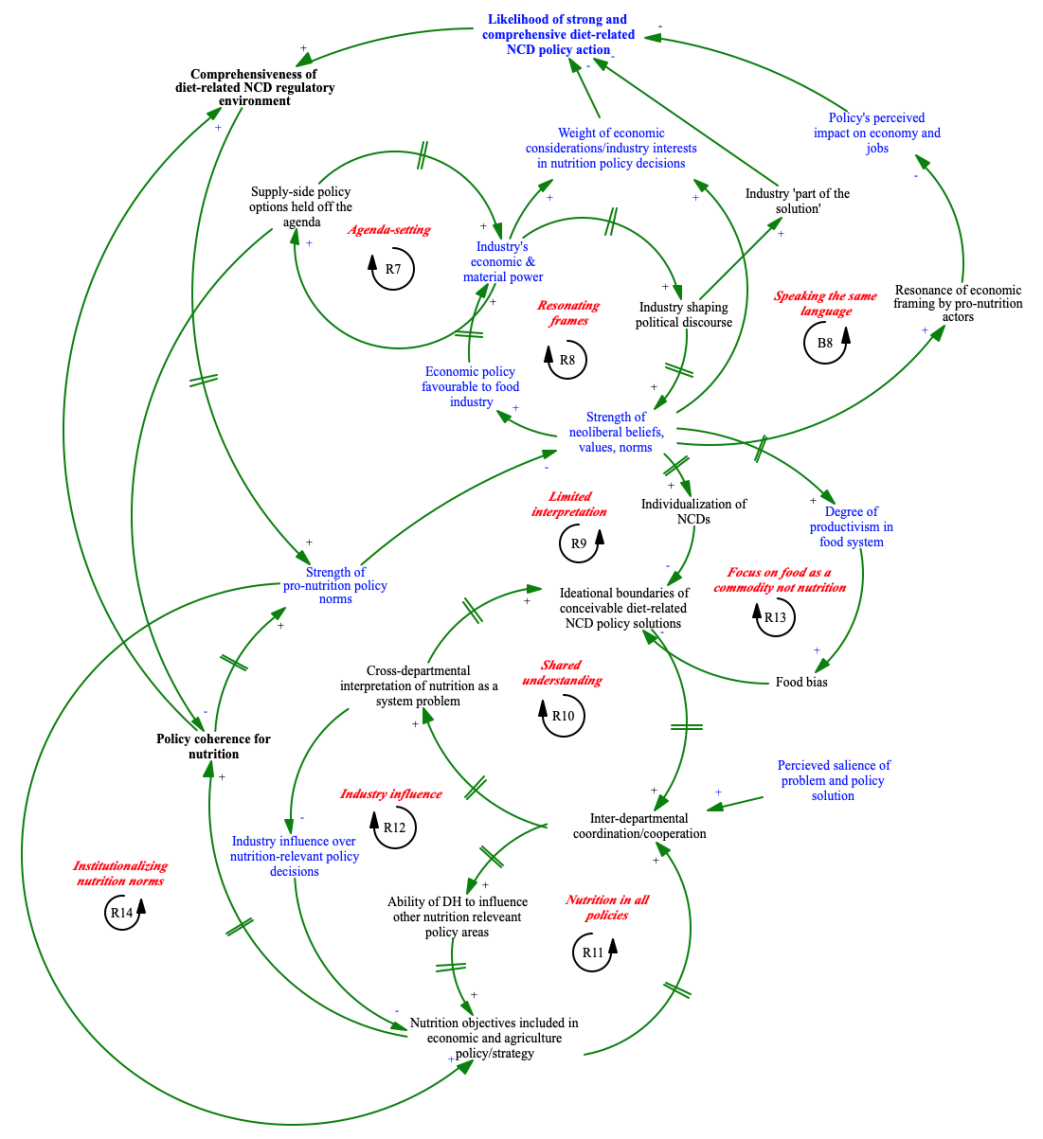
Sub-system II: Structural and Discursive Power.

 Sub-system II (Figure 2) illustrates stakeholders’ understanding of the interactions between various mechanisms of structural and discursive power (see Supplementary file 1). These mechanisms include ideologies, values, perception and preference shaping, organizational structures, and norms.^23^

*R7 Agenda-setting:* The economic power of the food industry prevents less powerful policy actors (eg, public health academics) from being able to promote on to the agenda of viable solutions, policy options that would have significant economic impacts on industry, including for example trade and investment policy levers. This is an aspect of industry’s structural power. This, in turn, allows the economic power of industry to continue to grow.

*B8 Speaking the same language:* Health policy-makers’ use of economic analytical tools (eg, costing analyses) and framing nutrition problems and policy solutions in economic terms can increase the likelihood of DR-NCD policy adoption by building broad support from more powerful economic policy actors. This can increase the comprehensiveness of the policy environment and strengthen pro-nutrition policy norms. Being a balancing loop indicates that using economic framing will only maintain norms at a steady state, not entirely transcend them.

*R8 Resonating frames:* Increased economic and material power of the food industry expands the capacity of industry to use various tactics (eg, issue framing and narratives communicated through corporate networks and the media as well as through privileged access to policy-makers) to shape the political discourse, looping back to reinforce the policy norms they tend to benefit from.

*R9 Limited interpretation:* The individualization of NCDs, where risk exposure is considered personal responsibility, not determined by complex structural drivers, is a natural extension of a socio-political context dominated by the imperative for economic growth. This interpretation tends to limit stakeholders’ ideational boundaries of conceivable policy solutions, ultimately weakening pro-nutrition policy norms and further strengthening the focus on economic growth. R9 therefore suggests that while policy actors can have agency over discursive power, it can also be deterministically generated from socio-political-economic system dynamics. Both R8 and R9 describe the dynamics of industry’s potential discursive (invisible) power.

*R10 Shared understanding:* Stakeholders reflected that, from their experience with access to medicines, institutional mechanisms can potentially contribute to disrupting industry discursive power. R10 illustrates that institutional structures and arrangements that increase inter-departmental co-ordination and cooperation may subsequently increase the capacity across departments to interpret and understand DR-NCDs as products of complex structural drivers across a range of sectors which, in turn, may expand stakeholders’ ideational boundaries of possible nutrition policy solutions, motivating further inter-departmental co-ordination and co-operation.

*R11 Nutrition in all policies:* Reducing policy-making silos and improving co-ordination between departments in economic policy development, can increase the DH’s influence within other policy domains including trade and investment and agriculture and lead to the inclusion of nutrition objectives in economic and agricultural policy and strategies, again increasing the desire for deeper inter-departmental coordination.

*R12 Industry influence: *Increased inter-departmental co-operation and collaboration can drive a shared understanding across government departments of nutrition as a system problem requiring a trans-sectoral approach. In turn this can weaken industry influence over nutrition-relevant policy decisions and promote the inclusion of nutrition objectives in economic and agriculture strategy/policy.

*R13 Focus on food as a commodity not nutrition:* Discursive power can be deterministically-driven creating policy environments that support the production of crops and food products (particularly ‘value-added’ products like UPFs) that maximize profit and their exportability. This normative approach to trade, investment and agricultural policy tends to drive ‘food bias’ where there is a perception, as one stakeholder reflected, that if there is sufficient food in the system, then the system ‘works,’ holding policies that would increase the nutritional quality of food, outside the boundaries of conceivable policy solutions. In turn, this contributes to poor policy coherence for nutrition across sectors and weakening of pro-nutrition policy norms over time which, without an alternative approach, strengthens the existing normative approach.

*R14 Institutionalizing nutrition norms:* Not including nutrition objectives in the overarching strategies of other nutrition-relevant policy areas including trade, investment and agriculture, leads to poor policy coherence for nutrition, weakening pro-nutrition policy norms and in turn further limiting the consideration of nutrition objectives across sectors. Non-health policy actors, for example, frequently cited that their mandate was to fulfil the economic and social development objectives laid out in the National Development Plan (NDP) however there is a significant lack of coherence between the NDP and nutrition policy. NDP objectives include increasing economic productivity and employment through agriculture, food processing and food retail and while food security is a key priority there is no mention of improving the nutritional quality of food.^49^ Economic strategy/policy documents like the NDP set the economic strategy for a determined period (eg, 5 years) and generate a cascading effect shaping the objectives across government departments and the performance reviews of their appointed officials and employees generating a ‘bureaucratic inertia’ as described by one stakeholder.

## Discussion of Policy Implications

 This work has used system dynamics methods to develop an initial complex system model of DR-NCD policy-making in South Africa mapping the inter-dependent political economy mechanisms that drive different forms of corporate power in the policy-making process. A growing body of research uses system dynamics or system mapping to better understand complex public health problems (and assess the effectiveness of alternative policy options), eg, obesity and other forms of malnutrition,^13,50,51^ inequities in healthy eating,^34^ tobacco control,^52-54^ NCDs,^55,56^ neonatal mortality,^57^ and the social determinants of health.^58^ However, just a few studies have used system dynamics methods to explore the politics of health (or specifically) food policy-making.^21,33^ Building on these, we suggest this work further illustrates the utility of using qualitative system mapping that visualises how causal factors are linked to each other in inter-dependent feedback processes. By revealing how an intervention affects both close and distant parts of the system, system mapping can provide deeper insight which strategies/interventions may be most useful for promoting more progressive and cohesive DR-NCD prevention policy.

 While it is not possible to reliably infer the short or long term impacts of interventions based on the system map alone, in this section we discuss some of the early strategic insights gained from this research. Adopting a systems thinking approach we discuss key feedback processes and potential leverage points in the system map developed in this work. We also consider how key interventions that have surfaced through analysing the system map as well as recommendations for driving NCD policy action identified by the Lancet Commission on Obesity, Undernutrition and Climate Change would impact on the DR-NCD policy-making system.^13^

 System actors tend to think and act in response to short causal chains and are generally insensitive to the presence of feedback between their decisions and the environment.^30^ However, by understanding feedback relationships within the system, and particularly reinforcing loops, potentially powerful leverage points can be identified^30^ that may shift power dynamics and promote DR-NCD policy action. The first key barrier-generating feedback processes identified in our system model, R1 in Sub-system I and R7 in sub-system II, reflect archetypal ‘success to the successful’ (or in this case, power to the powerful) systems traps.^30^ Weakening these feedback processes that expand the economic and material power of UPF-producing and selling corporations over time, may be important strategies to promote DR-NCD policy action. Strategies here might include, for example, embedding a framework and objectives for nutrition based on the World Health Organization (WHO) NCD Global Action Plan, within the remit of national and regional trade bodies eg, the South African Development Community^49^ and adopting regulations that internalize the true cost of food corporations’ products in terms of health and environmental impacts. In wealthy producing and exporting countries and regions the removal of perverse agricultural subsidies will likely be important eg, European Union subsidies on sugar.

 Other key barrier-generating feedback processes in sub-system I relate to nutrition policy actors’ adherence to strict evidence-based policy-making in response to industry pressure and in an effort to avoid legal disputes. However, considering more distal linkages within sub-system I indicates that an evidence-based approach can ultimately maintain weak pro-nutrition policy norms. This work therefore supports calls for an ‘evidence-informed and practice-based’ approach^59,60^ to DR-NCD policy decisions that promotes active policy experimentation and evaluation, since it could break the undesirable feedback loop described, ultimately potentially strengthening pro-nutrition policy norms with various positive repercussions across the system including weakening the individualization of NCDs to expand ideational boundaries of policy solutions and greater policy coherence for nutrition across sectors.

 Strengthening a number of existing facilitative feedback loops within sub-system I system may also be important to drive DR-NCD policy action and coherence. These include R6 *nutrition network mobilization*. In recent work Baker et al used system mapping methods providing a more detailed analysis of how nutrition networks may be strengthened to promote political commitment for malnutrition.^21^

 Driving a number of reinforcing loops in sub-system II including R10 *shared understanding*, R11 *nutrition in all policies*, R12 *reducing industry influence* and R14 *institutionalizing nutrition norms* will be important for overcoming the critical problem of nutrition not having a single departmental ‘home’ and, as a result, not being prioritized. Driving these loops could be important for promoting policy coherence across sectors towards generating a healthy and sustainable food system and reducing DR-NCDs. Key potential leverage points here include capacity building within the DH, DTI and DAFF and governance structures that ensure nutrition policy-makers are included in the development of trade and investment strategy and on negotiating teams. These interventions could make embedding a framework and objectives for nutrition within the remit of trade decision-making bodies, as suggested earlier, and including nutrition objectives within other key economic policy documents (eg, the NDP) more possible, in turn strengthening policy coherence and pro-nutrition policy norms.

 Another key leverage point is adding system structures that provides informational feedback where it was previously lacking, in other words making actors directly accountable for their own actions.^30^ These can be hard to implement where they require those in power to agree to being more accountable. Nonetheless, they might include for example, making the DTI pay directly out of their own budget for the healthcare costs of people requiring chronic management of DR-NCDs due to their economic policies and strategic decisions that increase availability and consumption of UPFs. This could contribute to driving R11 *nutrition in all policies *(sub-system II). Internalizing the cost of the health impacts of industry products would be another example which may have significant effects throughout the system.

 The rules of any system determine its scope and degrees of freedom (the number of ways the system can vary) and their adjustment can present high-leverage interventions.^30^ Rules include laws (strongest), punishments, incentives and informal social agreements (weakest). Given the power of the rules governing a system, it is highly concerning as is illustrated in our complex system map and in related work,^23^ that the food industry (and other corporations) has significant influence over the rules of international trade and nutrition policy at both the domestic and international level. It is industry’s shaping of trade rules that has helped unleash the ‘success to the successful’ loops leading to accumulation of industry productive power earlier described.

 A key rule in the nutrition policy-making system is the South African Constitutional requirement that policy-makers engage with all interested stakeholders during policy development, including industry. This rule alone limits the scope for pushing the system towards reducing food industry involvement in DR-NCD-relevant policy processes. However, the Constitution also commits the government to comply with its international obligations. This includes international health instruments like the Framework Convention on Tobacco Control which, under Article 5.3, obligates parties to adopt measures that protect “their public health policies related to tobacco control from commercial and other vested interests of the tobacco industry.”^61^ As such, systems thinking perspective may support proposals for a Framework Convention on Food Systems.^13^ This would drive sub-system I loop B2 *international instruments limiting industry engagement*, controlling R5 *industry legitimacy* to limit food industry participation and influence in policy development. The knock-on effects of such an instrument could powerfully facilitate policy action and promote pro-nutrition policy norms. That said, it may lead industry to adapt by strengthening more covert strategies to influence policy – including mechanisms of discursive power (eg, perception shaping through issue framing/narratives communicated through corporate foundations, opinion leaders and media capture).

 The overall goal or purpose of the system is one of the most powerful points of leverage in any system. Seeking the wrong goal will drive the system in an undesirable direction. For example, the goal of gross domestic product growth or economic growth more broadly, has been found to generate problems of unemployment, poverty, hunger, resource depletion, and environmental degradation.^62^ Arguably, the nutrition policy-making system presented in this work is not driven by an overarching goal of ensuring a nutritious, sustainable and equitable food system, but more by food security objectives and by the goal of expanding food corporations’ global market share for the sake of economic growth. System goals, along with its rules and relationships arise from core underlying paradigms – deeply held beliefs and associated assumptions about how the world works.^30^ During this research we identified a number of paradigmatic assumptions including ‘consumption-based growth is critical for development,’ ‘the food system is a resource to be converted to economic gain’ and ‘trade is ultimately good for health.’ As the system’s source, intervening at the level of the paradigm (in this case neoliberalism) can be transformative. A systems perspective therefore strongly supports increasing calls from the public health and new economics communities for a new paradigm that seeks to meet the health and social needs of the population within the means of the planet.^63^ Paradigm shifts is a field of research in itself but broadly requires persistently highlighting failures of the existing paradigm, framing problems, challenges and solutions according to the new one, positioning advocates of the new paradigm in positions of power and visibility, and focusing on building broad support.^30^

 Finally, it is important to note that while we’ve suggested a number of potential policy/strategic recommendations and their possible impacts, systems are highly resilient and actors within them will often respond with efforts to undermine system change (and maintain existing power relations). For example, industry may respond to a tax on sugar-sweetened beverages by introducing a low-sugar version alongside their regular product, increasing brand visibility, recognition and sales,^64^ indicating taxes may in fact do little to reduce the economic power of the food industry (or NCDs). Careful consideration of how system actors’ responses may undermine the intended effects of the interventions proposed in this section is therefore essential. Additionally, the feasibility of a number of the interventions outlined would require further consideration of other political economy factors described in the model.

###  Limitations

 Important variables, links and feedback structures may not have been captured in the model for a number of reasons. Firstly, given system dynamic models are constructed based primarily on stakeholders’ understanding of the problem under investigation (and in this research, the addition of a small number of causal linkages from two literature reviews) it is quite possible the model contains inaccuracies and deficiencies due to stakeholder subjectivity and limited understanding.^65^ While significant effort was made to include as many stakeholders as possible with intimate understanding of different aspects of the problem, it was challenging to access high level politicians/government officials who may have provided additional system insights. Second, due to the political nature of the research topic and the inherent vested interest of different stakeholders, it is possible some participants were sharing politically motivated reasoning or omitting certain casual relationships from discussion in the interviews. Failing to capture important variables is likely to have affected the reliability and depth of the insights gained.

 While having only the primary researcher (PM) conduct the PTA and model development ensured consistent coding it also introduces the risk of potential bias. However, this risk was reduced by having the same researcher also collect the data. This provided the opportunity for asking probing questions to gain a deeper understanding of the context of the data at the time of data collection^66^ which facilitated response to “subtle nuances of, and cues to, meaning in the data” during data analysis^67^ to reduce potential bias. A key to step to mitigate modeler bias was conducting follow-up model validation discussion sessions with a sub-set of stakeholders.^66^

 Generalizability to other country contexts is limited given the context-specific nature of the problem under investigation. A next step would be to engage stakeholders in another country context to explore similarities and differences in the causal structure of the South African conceptual model.

 Finally, the conceptual model alone provides only preliminary insight into what actions might promote more progressive and coherent NCD prevention policy over time. We consider developing and experimenting with a numerical simulation model as important for confirming understanding of the system structure and testing the effectiveness of different interventions over time. As such, future work could involve proceeding to simulation modelling to further validate the system structure, understand the comparative strengths of the different feedback loops, how the systems behaviour changes over time and which interventions would be most effective in promoting more progressive DR-NCD policy in the long run. That said, this work does represent the first attempt to conceptualize DR-NCD policy inaction as a complex systems problem and to qualitatively map the causal structure of the problem by defining the feedback relationships between elements in CLDs.^68^ We argue this does have stand alone utility for informing future thinking about how to promote healthy policy action. Supplementary file 5 provides reflexivity considerations.

## Conclusion

 Using qualitative system dynamic modelling we developed a complex system model for understanding DR-NCD policy inaction due to political economy mechanisms and corporate power. This work demonstrates the utility in adopting a systems thinking approach that visualises the inter-dependence and feedback processes between different factors that drive patterns in health policy-making over time. Better understanding the complexity in health policy-making through use of complex system methods can facilitate the identification and more insightful evaluation of potential strategies for promoting more progressive health policy-making. Future work should explore the possibility of proceeding to quantitative simulation modelling to evaluate the dynamic short and long-term impacts of different strategies on the political economy and power dynamics in DR-NCD policy-making. A key benefit of using a systems thinking approach in future work (and to encourage ‘system doing’) may be to bring government stakeholders together in a group model building exercise to build a shared ‘systems’ understanding of the interconnectedness of drivers of not only NCDs themselves but also the observed pattern of policy inaction to effectively prevent NCDs. Doing so may facilitate non-health actors to see their own role in the system promoting greater inter-departmental co-operation to prevent DR-NCDs.

## Ethical issues

 Participants provided written informed consent to participate in this study. Ethical approval for this research was granted by both the University of Cape Town’s Human Research Ethics Committee and the London School of Hygiene and Tropical Medicine’s Research Ethics Committee.

## Competing interests

 Simon Moeketsi Modisenyane was working for the South African Department of Health at the time of this research.

## Funding

 This work was supported by funding from the Wellcome Trust [203286/Z/16/Z] to support this research.

## Supplementary files


Supplementary file 1. The Conceptual Framework for Analysing Power in Public Health Policy-Making.

Supplementary file 2. Example of Purposive Text Analysis Coding Chart.

Supplementary file 3. Causal Loop Diagram Combination.

Supplementary file 4. Pre-validation Shared Mental Model.

Supplementary file 5. Reflexivity.
