# Supplementary material for: Using System Dynamics to Understand Transnational Corporate Power in Diet-Related Non-communicable Disease Prevention Policy-Making: A Case Study of South Africa
Source: Int J Health Policy Manag. 2023 Sep 17;12:7641. doi: 10.34172/ijhpm.2023.7641 (PMC10590239; doi:10.34172/ijhpm.2023.7641)
Supplement: Supplementary file 1 — The Conceptual Framework for Analysing Power in Public Health Policy-Making. [file ijhpm-12-7641-s001.pdf]

Article title: Using System Dynamics to Understand Transnational Corporate Power in Diet-Related Non-communicable Disease Prevention Policy-Making: A Case Study of South Africa
Journal name: International Journal of Health Policy and Management (IJHPM)
Authors' information: Penelope Milsom[1]*, Andrada Tomoaia-Cotisel[2], Richard Smith[3], Simon Moeketsi Modisenyane[1], Helen Walls[1]
[1]Department of Global Health and Development, Faculty of Public Health and Policy, London School of Hygiene and Tropical Medicine, London, UK.
[2]RAND Corporation, Santa Monica, CA, USA.
[3]College of Medicine and Health, University of Exeter, Exeter, UK.
***Correspondence to**: Penelope Milsom, Email: Penelope.milsom@lshtm.ac.uk
Citation: Milsom P, Tomoaia-Cotisel A, Smith R, Modisenyane SM, Walls H. Using system dynamics to understand transnational corporate power in diet-related non-communicable disease prevention policy-making: a case study of South Africa. Int J Health Policy Manag. 2023;12:7641. doi:10.34172/ijhpm.2023.7641
Supplementary file 1. The Conceptual Framework for Analysing Power in Public Health Policy-Making

This framework was first published in Milsom et al, 2020 (1) and was developed for use as a heuristic for more deeply understanding *how* different forms of power are expressed via multiple inter-related mechanisms (operating in different spaces and across levels) to influence health policymaking.

Figure 1: Conceptual framework for analysing power in public health policymaking

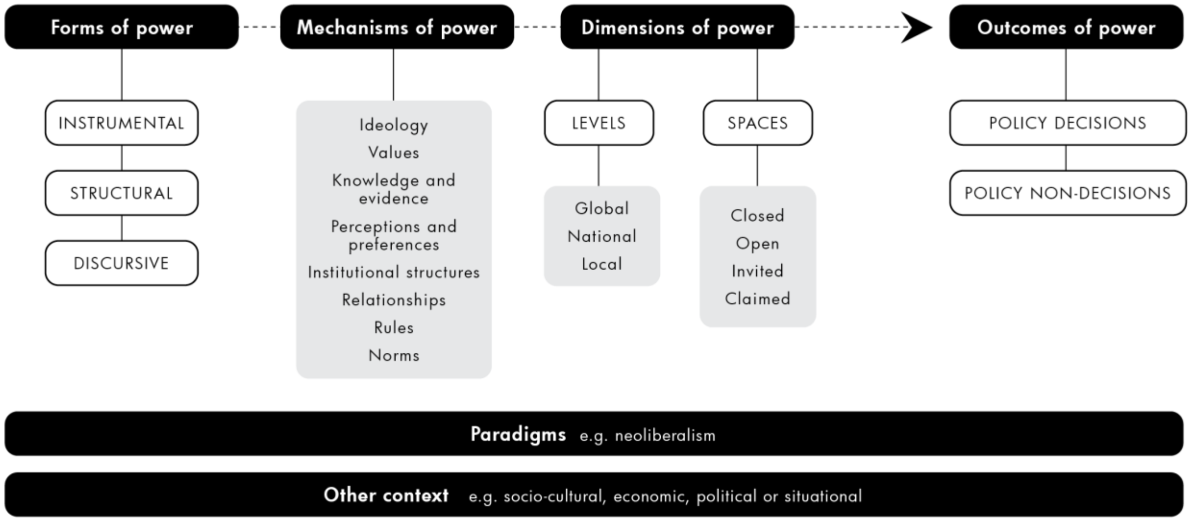

The *forms* of power described in the conceptual framework are derived from Fuchs and Lederer's framework(2) and heavily influenced by Lukes' Three Dimensions of Power (3). Instrumental power is defined as the direct influence different stakeholders have over formal policymakers' voluntary decisions. Structural power refers primarily to agenda-setting power- the ability to limit who is included at the table, whose interests are prioritised and the scope of alternatives being considered. Discursive power involves shaping perception and interpretation of problems such that potentially effective solutions are held outside the minds of stakeholders. Discursive power usually results from the combination of both deliberate action and structural processes of socialization and internalization of accepted paradigms/ideologies (1, 4)

The framework proposes that each *form* of power can be expressed via eight interdependent *mechanism* types as outlined in Figure 1. adapted from the 'Three Is' framework (5-8) and with examples drawn from Madureira Lima and Galea's framework of corporate practices and health (9). These are ideologies(e.g. the neoliberal political 'project'); values (e.g., individual freedom and choice); knowledge and evidence (e.g., manufacturing doubt); perception and preference-shaping (e.g. via issue framing and narratives); organisational structures (e.g., corporate participation in government committees and commissions); relationships (e.g., corporate lobbying); rules (e.g., trade agreements and investment treaties); and norms (e.g., prioritization of economic growth over health in political decision-making) (1).

Mechanisms are active in different spaces (closed, open, invited, claimed) and at different levels (international, national or sub-national) as described in Gaventa's Power Cube (10). *Spaces* are formal or informal opportunities where actors can 'potentially affect policies, discourses, decisions and relationships' relevant to their interests (10).

The *outcome* of power in health policymaking may be either a *policy decision* taken by decision-makers to act (voluntary/involuntary and optimally/sub-optimally) (1) or a *non-decision (*a voluntary decision not to act/an involuntary failure to act/ inaction due to an ideational boundaries issue) (1). Different contexts – political, economic, socio-cultural or situational – influence which mechanisms are active and effective in a policymaking process (1). Overarching *paradigms* determine the overall structure of power in the policymaking system (1).

References

1. Milsom P, Smith R, Baker P, Walls H. Corporate power and the international trade regime as drivers of NCD policy inactions: A realist review Health Policy and Planning. 2020.
2. Fuchs D, Lederer M. The power of business. Business in Politics. 2007;9(3).

3. Lukes S. Power: A Radical Approach. London: McMillan; 1974.
4. Milsom P, Smith R, Modisenyane SM, Walls H. Does international trade and investment liberalization facilitate corporate power in nutrition and alcohol policymaking? Applying an integrated political economy and power analysis approach to a case study of South Africa. Global Health. 2022;18(1):32.
5. Hall A. The role of interests, institutions, and ideas in the comparative political economy of the industrialized nations. In: Lichbach I, Zuckerman AS, editors. Comparative politics: Rationality, culture, and structure. Cambridge: Cambridge University Press; 1997. p. 174-207.
6. Gauvin F-P. Understanding Policy Developments and Choices Through the “3-i” Framework: Interests, Ideas and Institutions. Briefing Note2014.
7. Lavis JN, Ross SE, Hurley J, J.M H, Stoddart GL, Woodward CA, et al. Examining the role of health services research in public policymaking. Milbank Quarterly. 2002;80(1):125-54.
8. Shearer JC, Abelson J, Kouyate B, Lavis JN, Walt G. Why do policies change? Institutions, interests, ideas and networks in three cases of policy reform. Health Policy Plan. 2016;31(9):1200-11.
9. Madureira Lima J, Galea S. Corporate practices and health: a framework and mechanisms. Global Health. 2018;14(1):21.
10. Gaventa J. Finding the spaces for change: A power analysis. IDS Bulletin. 2006;37(6).
