# Supplementary material for: Using System Dynamics to Understand Transnational Corporate Power in Diet-Related Non-communicable Disease Prevention Policy-Making: A Case Study of South Africa
Source: Int J Health Policy Manag. 2023 Sep 17;12:7641. doi: 10.34172/ijhpm.2023.7641 (PMC10590239; doi:10.34172/ijhpm.2023.7641)
Supplement: Supplementary file 2 — Example of Purposive Text Analysis Coding Chart. [file ijhpm-12-7641-s002.pdf]

Article title: Using System Dynamics to Understand Transnational Corporate Power in Diet-Related Non-communicable Disease Prevention Policy-Making: A Case Study of South Africa

Journal name: International Journal of Health Policy and Management (IJHPM)

Authors' information: Penelope Milsom[1]*, Andrada Tomoaia-Cotisel[2], Richard Smith[3], Simon Moeketsi Modisenyane[1], Helen Walls[1]

[1]Department of Global Health and Development, Faculty of Public Health and Policy, London School of Hygiene and Tropical Medicine, London, UK.

[2]RAND Corporation, Santa Monica, CA, USA.

[3]College of Medicine and Health, University of Exeter, Exeter, UK.

***Correspondence to**: Penelope Milsom, Email: Penelope.milsom@lshtm.ac.uk

Citation: Milsom P, Tomoaia-Cotisel A, Smith R, Modisenyane SM, Walls H. Using system dynamics to understand transnational corporate power in diet-related non-communicable disease prevention policy-making: a case study of South Africa. Int J Health Policy Manag. 2023;12:7641. doi:10.34172/ijhpm.2023.7641

Supplementary file 2. Example of Purposive Text Analysis Coding Chart

Below is an exert of purposive text analysis of one of the stakeholder interviews using the coding chart(1). This figure illustrates how causal phrases are identified within stakeholder statements and translated into a causal or effect model variable. A corresponding words and arrow diagram is then developed illustrating the causal structure and the relationship type between each variable (positive or negative).

**Participant quotation:**	**Causal phrases requiring interpretation:**	**Model variable:**
<u>So the minute we start specifying certain things in a regulation [1]</u>, <u>the next product that come out, will be able to go through that</u>. So that's what we constantly also have to formulate in the legislation, is to say, we can't really list everything because they'll find something else that, like you'll call something a yoghurt. You can only use a yoghurt as in this, this, this. Now <u>lots of people eat yoghurt, but now</u>	*So the minute we start specifying certain things in a regulation*	**Public health regulation (causal)**
	the next product that come out, will be able to go through that.	**Product innovation (effect)**
	So that's what we constantly also have to formulate in the legislation	**Public health regulation (effect)**
	Lots of people eat yoghurt	**Consumption of unhealthy product (causal)**

<table>
<tr><td rowspan="4">you regulate it: what the composition must be, or whatever the scenario must be. Then they’ll come along with something called Yogetta or something, it’s for the consumer to know what it is, but it’s not actually yoghurt because they’re trying to get around legislation and the content</td><td>but now you regulate it: what the composition must be, or whatever the scenario must be</td><td>Public health regulation (effect)</td></tr>
<tr><td>they’ll come along with something called Yogetta or something, it’s for the consumer to know what it is, but it’s not actually yoghurt because they’re trying to get around legislation</td><td>Industry product innovation (effect)</td></tr>
<tr><td colspan="2">Main argument:</td></tr>
<tr><td colspan="2">When public health regulation is introduced in response to high consumption of an unhealthy product, the food industry is able to adapt their products in response, requiring further amendments to the regulation.</td></tr>
<tr><td colspan="3">Drawing of causal structure:</td></tr>
<tr><td colspan="3">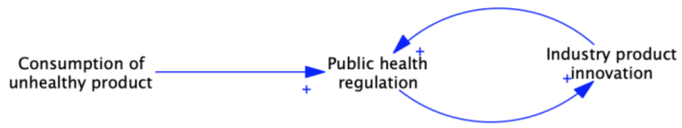
</td></tr>
</table>

References

1. Tomoaia-Cotisel A. The Journey toward the Patient-Centered Medical Home: A Grounded, Dynamic Theory of Primary Care Trans-formation. London: London School of Hygiene and Tropical Medicine; 2018.
