# Supplementary material for: Using System Dynamics to Understand Transnational Corporate Power in Diet-Related Non-communicable Disease Prevention Policy-Making: A Case Study of South Africa
Source: Int J Health Policy Manag. 2023 Sep 17;12:7641. doi: 10.34172/ijhpm.2023.7641 (PMC10590239; doi:10.34172/ijhpm.2023.7641)
Supplement: Supplementary file 3 — Causal Loop Diagram Combination. [file ijhpm-12-7641-s003.pdf]

Article title: Using System Dynamics to Understand Transnational Corporate Power in Diet-Related Non-communicable Disease Prevention Policy-Making: A Case Study of South Africa

Journal name: International Journal of Health Policy and Management (IJHPM)

Authors' information: Penelope Milsom[1]*, Andrada Tomoaia-Cotisel[2], Richard Smith[3], Simon Moeketsi Modisenyane[1], Helen Walls[1]

[1]Department of Global Health and Development, Faculty of Public Health and Policy, London School of Hygiene and Tropical Medicine, London, UK.

[2]RAND Corporation, Santa Monica, CA, USA.

[3]College of Medicine and Health, University of Exeter, Exeter, UK.

***Correspondence to**: Penelope Milsom, Email: Penelope.milsom@lshtm.ac.uk

Citation: Milsom P, Tomoaia-Cotisel A, Smith R, Modisenyane SM, Walls H. Using system dynamics to understand transnational corporate power in diet-related non-communicable disease prevention policy-making: a case study of South Africa. Int J Health Policy Manag. 2023;12:7641. doi:10.34172/ijhpm.2023.7641

Supplementary file 3. Causal Loop Diagram Combination

During causal loop diagram (CLD) combination a number of strategies were adopted. When two CLDs undergoing combination are entirely complimentary, a simple additive approach(1) was adopted as illustrated below in Figure 1. For example, if Stakeholder #1 identifies A→B→C→A and Stakeholder #2 identifies A→D→C→A, then the combined CLD would have all identified structures.

Figure 1. Additive CLD combination

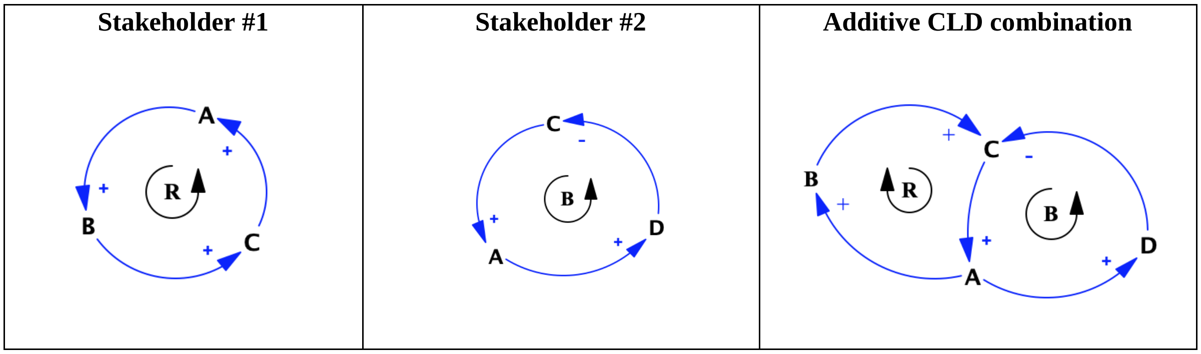

Two main approaches were adopted when CLD structures were not directly compatible and a judgement had to be made about which was the most accurate. The first approach was to select the most detailed description of the system structure(1) as shown in Figure 2. For example, if Stakeholder #3 identified the same causal structure as Stakeholder #1 (A→B→C) but with the inclusion of on addition of variable (E), then the more detailed causal description was included.

Figure 2. Selection of most detailed CLD

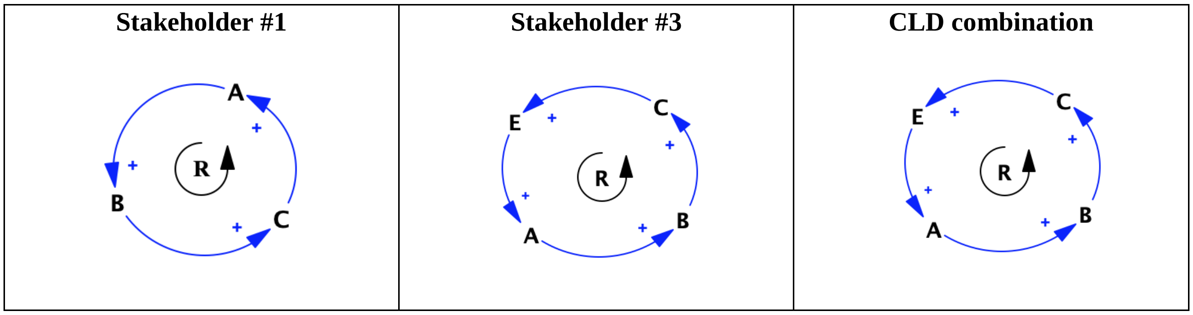

In a number of instances stakeholders described the same phenomenon using different language or scenarios which added significant complexity to the CLDs reducing their practical usability while not adding to dynamic complexity (1). In these situations we adopted a second technique whereby variables describing different examples of the same phenomena were combined under one more generalized variable at a higher level of abstraction(1, 2). For example, in Figure 3. Stakeholder #4 describes F, G and H and Stakeholder #5 describes I and J which are all examples of the more general term C so the CLDs are combined and simplified to A→B→C→A.

Figure 3. Merging and generalizing variables

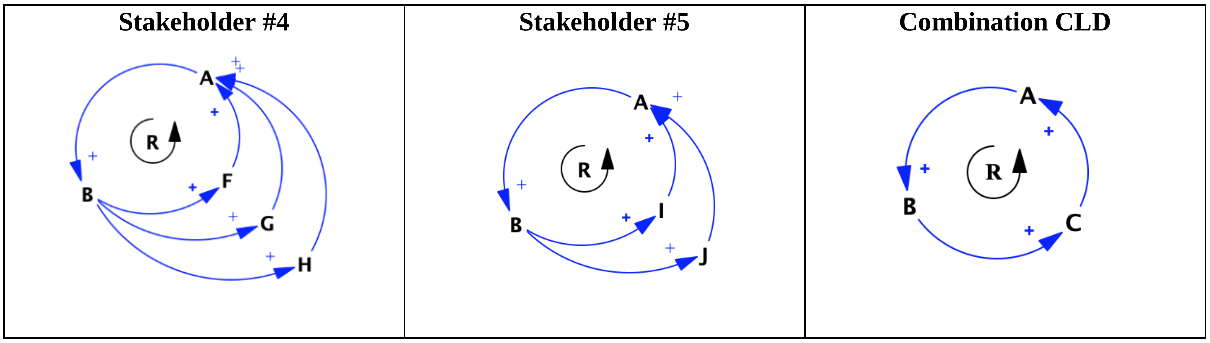

Where F, G and H are different scenarios or language describing C	Where I and J are different scenarios or language describing C	Where B is a more general term capturing F, G, H, I and J

At times during initial inidivudal CLD development and again in the final stage of shared CLD development, causal relationships were either so obvious that stakeholders did not mention them or they were somewhat more subtly implied by the context. In these instances the implied causal structure was also included in the CLD. For example if Stakeholder #6 identifies K→L→K but the obvious link to M is not mentioned, the final CLD would be K→L→M→K. Decomposing CLDs was considered acceptable given that the same interviewee (PM) also conducted the purposive text analysis and CLD combination, which facilitated sensitivity to “subtle nuances of, and cues to, meaning in the data” during data analysis(3).

Figure 4. Decomposing CLDs

**Stakeholder #6**	**Decomposed CLD**
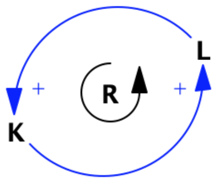	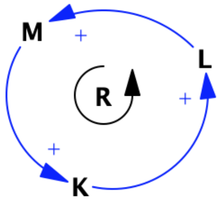 Where M is implied by the context of the discussion topic

References

1. Tomoaia-Cotisel A. The Journey toward the Patient-Centered Medical Home: A Grounded, Dynamic Theory of Primary Care Trans-formation. London: London School of Hygiene and Tropical Medicine; 2018.
2. Kim H, Anderson DF. Building confidence in causal maps generated from purposive text data: Mapping transcripts of the Federal Reserve. System Dynamics Review. 2012;28(4).
3. Strauss AL, Corbin JM. Basics of Qualitative Research: Techniques and Procedures for Developing Grounded Theory (2nd edn). Thousands Oaks, California: Sage Publications; 1998.
