# Supplementary material for: Using System Dynamics to Understand Transnational Corporate Power in Diet-Related Non-communicable Disease Prevention Policy-Making: A Case Study of South Africa
Source: Int J Health Policy Manag. 2023 Sep 17;12:7641. doi: 10.34172/ijhpm.2023.7641 (PMC10590239; doi:10.34172/ijhpm.2023.7641)
Supplement: Supplementary file 4 — Pre-validation Shared Mental Model. [file ijhpm-12-7641-s004.pdf]

Article title: Using System Dynamics to Understand Transnational Corporate Power in Diet-Related Non-communicable Disease Prevention Policy-Making: A Case Study of South Africa

Journal name: International Journal of Health Policy and Management (IJHPM)

Authors' information: Penelope Milsom[1]*, Andrada Tomoaia-Cotisel[2], Richard Smith[3], Simon Moeketsi Modisenyane[1], Helen Walls[1]

[1]Department of Global Health and Development, Faculty of Public Health and Policy, London School of Hygiene and Tropical Medicine, London, UK.

[2]RAND Corporation, Santa Monica, CA, USA.

[3]College of Medicine and Health, University of Exeter, Exeter, UK.

***Correspondence to**: Penelope Milsom, Email: Penelope.milsom@lshtm.ac.uk

Citation: Milsom P, Tomoaia-Cotisel A, Smith R, Modisenyane SM, Walls H. Using system dynamics to understand transnational corporate power in diet-related non-communicable disease prevention policy-making: a case study of South Africa. Int J Health Policy Manag. 2023;12:7641. doi:10.34172/ijhpm.2023.7641

Supplementary file 4. Pre-validation Shared Mental Model

Variables and links that were identified from the stakeholder interviews are coloured black and green respectively. Variables or links included in the SMM based on findings of the previously conducted realist review are presented as purple variables and dashed purple lines in the sub-sections of the pre-validation SMM below. If these variables and/or links were validated by stakeholders during the validation discussions, they were converted to black variables and green links in the final conceptual model presented in the paper. Variables or links that were added to the model or re-conceptualized as a result of the validation interviews are presented here in pink. As for the conceptual model, variables linking to the other sub-system are blue and variables only within a single sub-system are black. Names of feedback loops are in red.

Sub-System I: Instrumental power

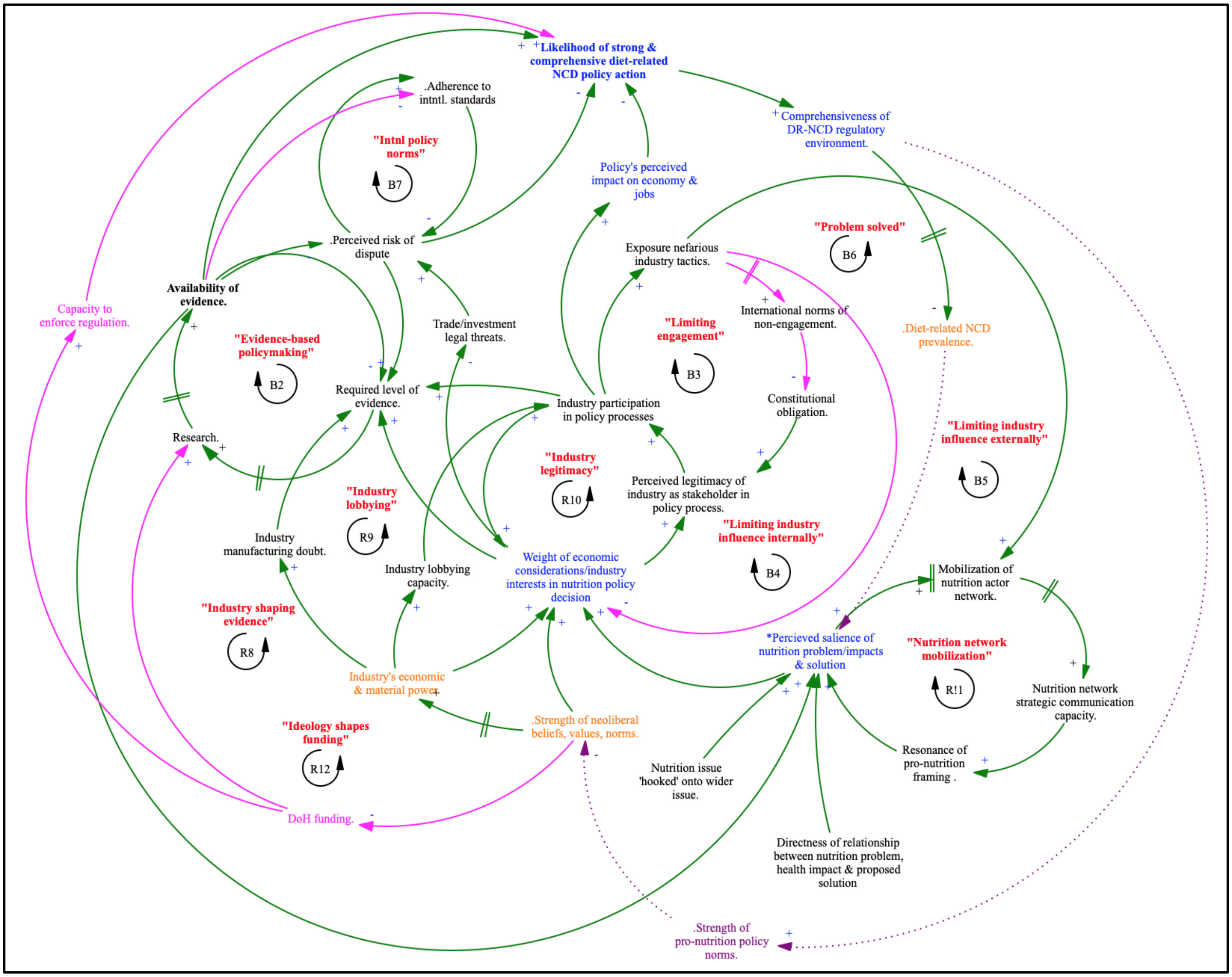

Sub-System III: Structural and discursive power

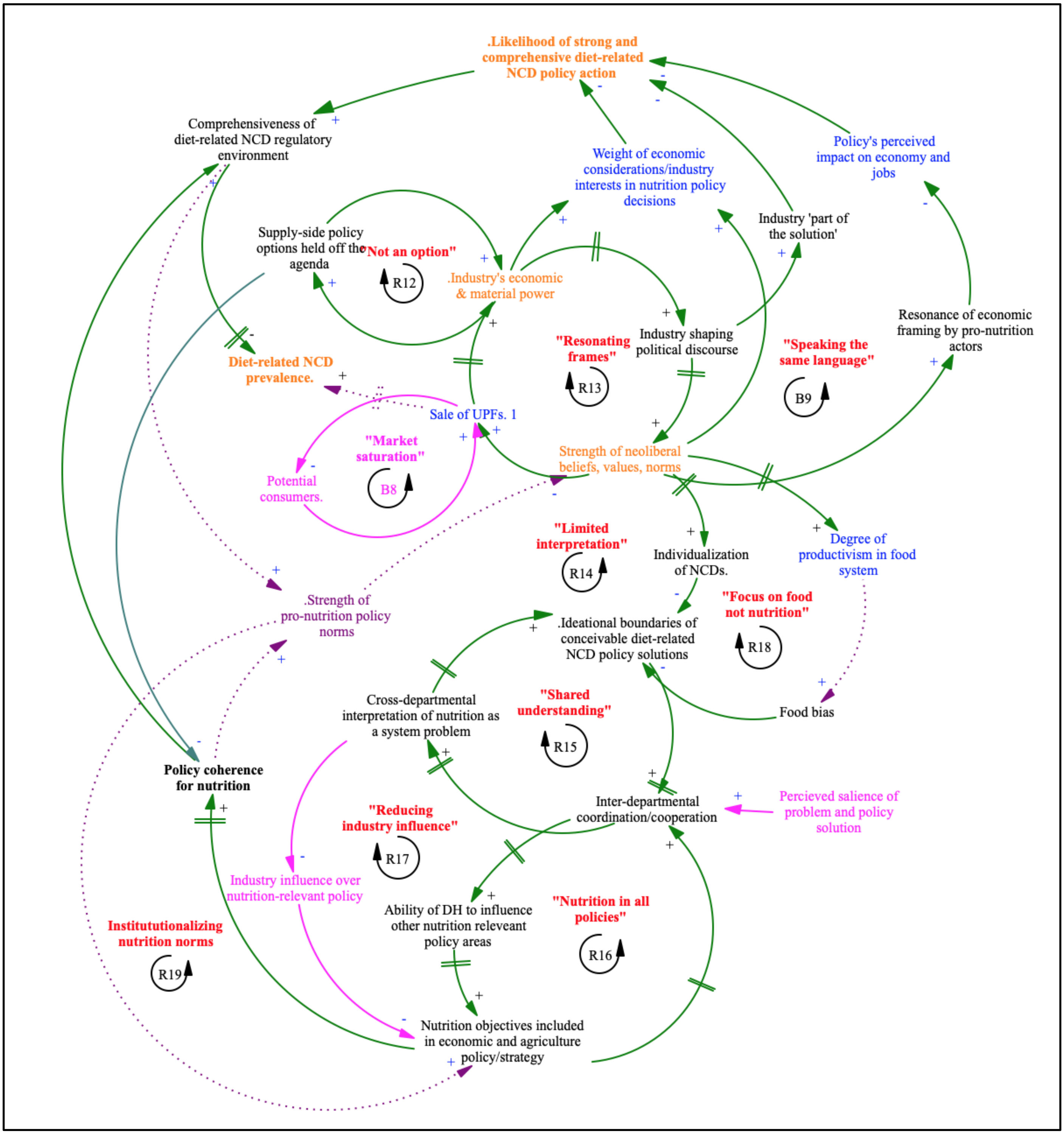

Key

Arrows indicate the direction of the influence	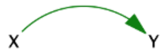
Positive polarity indicates that the influencing variable and the receiving variable change in the same direction	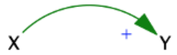
Negative polarity indicates the receiving variable changes in the opposite direction of the influencing variable	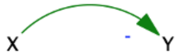
Delay between cause and effect	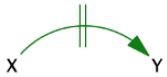
Reinforcing loop	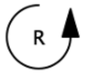
Balancing loop	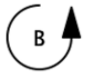
