# Supplementary material for: Using System Dynamics to Understand Transnational Corporate Power in Diet-Related Non-communicable Disease Prevention Policy-Making: A Case Study of South Africa
Source: Int J Health Policy Manag. 2023 Sep 17;12:7641. doi: 10.34172/ijhpm.2023.7641 (PMC10590239; doi:10.34172/ijhpm.2023.7641)
Supplement: Supplementary file 5 — Reflexivity. [file ijhpm-12-7641-s005.pdf]

Article title: Using System Dynamics to Understand Transnational Corporate Power in Diet-Related Non-communicable Disease Prevention Policy-Making: A Case Study of South Africa

Journal name: International Journal of Health Policy and Management (IJHPM)

Authors' information: Penelope Milsom[1]*, Andrada Tomoaia-Cotisel[2], Richard Smith[3], Simon Moeketsi Modisenyane[1], Helen Walls[1]

[1]Department of Global Health and Development, Faculty of Public Health and Policy, London School of Hygiene and Tropical Medicine, London, UK.

[2]RAND Corporation, Santa Monica, CA, USA.

[3]College of Medicine and Health, University of Exeter, Exeter, UK.

***Correspondence to**: Penelope Milsom, Email: Penelope.milsom@lshtm.ac.uk

Citation: Milsom P, Tomoaia-Cotisel A, Smith R, Modisenyane SM, Walls H. Using system dynamics to understand transnational corporate power in diet-related non-communicable disease prevention policy-making: a case study of South Africa. Int J Health Policy Manag. 2023;12:7641. doi:10.34172/ijhpm.2023.7641

Supplementary file 5. Reflexivity.

Reflexivity is defined as 'recognizing that the researcher is part of the process of producing the data and their meanings, and a conscious reflection on that process'(1). This supplementary text therefore contains a conscious reflection on how the primary investigator's (PI) backgrounds and beliefs might have entered their qualitative research practice (2), while acknowledging that it is not possible to be aware of many of the subconscious ways in which our assumptions shape our research (3).

First, it is important to recognize that the research question in this work: how does the political economy of DR-NCD policymaking cause policy inertia (and how might this be addressed) was not initially developed from the 'bottom-up' by stakeholders in South Africa, but rather by the PI and her team based at a academic institution in a high-income country (HIC) with subsequent significant consultation and refinement through discussions and input from a number of key local stakeholders working within the South African government to ensure answering the research question would be of use to them. In future work we would, if at all possible, ensure the research question was, from the outset developed by local stakeholders. This is for ethical reasons, the crucial decolonizing global health imperative and to maximize the chance of stakeholder buy-in to participate and learn from the research which is particularly essential in system model building work since it requires a significant time commitment from stakeholders (participants) to build, validate then discuss the model and its implications.

The primary researcher's 'outsider' position as a public health researcher from a foreign research institution in a HIC primarily engaging with 'elite' policy stakeholders in South Africa likely affected to some extent the data collected in stakeholder interviews and used for model building. Firstly, this may well have affected who was willing to take part in the research. Multiple high-level 'elite' policymakers either declined or did not respond to invitations to participate in this research, indicating both their ability to protect themselves from intrusion and potentially also the outsider status of the PI. Other stakeholders may have ignored invitations to participate due to concerns that, as a public health researcher, the PI's interests may have been to be critical of them and their practices. Given the public health background of the PI, they were considered somewhat more of an 'insider' or at least an ally amongst some health policymakers as well as academics and NGO/IGOs, potentially making them more willing to find time in their busy schedules to take part in an interview.

Significant efforts were made to address the recognised challenges of recruiting 'elite' stakeholders (1, 4). These included by respecting their status and position (e.g. by referring to them by their titles), indicating knowledge of their background and expertise and outlining the unique and important contribution they would make to this research in correspondence; through introductions by respected 'insider' intermediaries; and by repeated follow-ups (in some cases up to six times) via email or phone. However, after six months of data collection we were successful in securing just one interview amongst the most elite stakeholders (Director Generals, Deputy Director Generals, Ministers or Health Attachés). Not having the opportunity to include more elite policymakers and politicians may well have limited the breadth of perspectives obtained on factors that affect health policy processes in South Africa and may mean that there are certain more political dimensions of the policymaking process missed in the complex system model developed. However, one insider commented that cabinet meetings are confidential and high-level policymakers and politicians are not at liberty to share confidential policy discussions nor would they be willing to disclose the content of informal private meetings, indicating that securing interviews with these stakeholders may not necessarily have provided significantly richer explanatory data.

Positionality may also have affected the data collected. Health policymakers as compared to trade/investment policymakers and other stakeholders tended to be somewhat more defensive in their responses during interviewing. This may have been partly due to the politically-sensitive nature of the research topic and the questions asked but potentially also their knowledge that I was of a public health background from a HIC public health research institution and therefore potentially came with a set of preconceived ideas and judgements about health policymaking in their country. For example, a health

policy during one model validation session asked ‘when will it be considered that we’ve done enough as health policymakers?’. As such, positionality may have restricted the information shared by some health policymakers. In contrast, trade/investment policymakers appeared relatively open during interviewing which may be due in part to the PI’s lack of authority as a public health researcher to judge their performance given their entirely non-health mandate with exclusively economic goals and performance indicators.

As an outsider the PI had the freedom to ask taboo or ‘obvious’ questions during interviews and possibly to elicit fuller explanations than perhaps an insider might have been able to. The PI also came with a ‘clean slate’ which some argue may have allowed for a more objective analysis of the policymaking process (46)(5). However, as mentioned, a number of challenges comes with being an ‘outsider’ including lack of access to stakeholders and having a less authentic understanding of the policy culture under study (46)(5). To manage these limitations, the PI worked relatively closely with a South African Department of Health official on this research team (MM) who assisted in shaping the research question and negotiating access to certain elite stakeholders and provided valuable additional historical and cultural context during regular meetings to discuss the research findings during the data collection phase. MM was also involved in reviewing the analysis to ensure my interpretation of the data was sufficiently contextualized. Despite MM’s valuable input, we acknowledge that with an ‘outsider’ PI conducting this research and lacking ‘closeness to the operational reality’ (6) of policymaking in South Africa, there remains a risk that the purposive text analysis and model conceptualization undertaken was somewhat ‘superficial and decontextualized’ (7), with the potential to affect the model’s validity. However, the model validation interviews with eight key stakeholders were conducted to minimize the risk of any researcher bias influencing the structure of the model.

Finally, the research team’s lack of previous experience using system dynamics methods may have affected the quality of the purposive text analysis and model conceptualization. To reduce this risk as far as possible the PI attended an intensive short course in system dynamics prior to commencing the research.

Reference

1. Green J, Thorogood N. Qualitative methods for health research. London, UK: SAGE Publications; 2013.
2. Richie J, Lewis J. Qualitative Research Practice: A guide for social science students and researchers. Richie J, Lewis J, editors. London: SAGE Publications 2003.
3. Seale C. The quality of qualitative research. London: Sage Publications; 1999.
4. Lui X. Interviewing elites: Methodological issues confronting a novice. International Journal of Qualitative Methods. 2018;17.

5. Merriam SB, Johnson-Bailey J, Lee MY, Kee Y, Ntseane G, Muhamad M. Power and positionality: negotiating insider/outsider status within and across cultures. International Journal of Lifelong Education.20.
6. Sheikh K, George A, Gilson L. People-centred science: strengthening the practice of health policy and systems research. Health Research Policy and Systems 2014;12(19).
7. Walt G, Shiffman J, Schneider H, Murray SF, Brugha R, Gilson L. 'Doing' health policy analysis: methodological and conceptual reflections and challenges. Health Policy Plan. 2008;23(5):308-17.
